# A Robust Brain Tumor Detector Using BiLSTM and Mayfly Optimization and Multi-Level Thresholding

**DOI:** 10.3390/biomedicines11061715

**Published:** 2023-06-15

**Authors:** Rabbia Mahum, Mohamed Sharaf, Haseeb Hassan, Lixin Liang, Bingding Huang

**Affiliations:** 1 Department of Computer Science, University of Engineering and Technology Taxila, Taxila 47050, Pakistan; 2 Industrial Engineering Department, College of Engineering, King Saud University, P.O. Box 800, Riyadh 11421, Saudi Arabia; mfsharaf@ksu.edu.sa; 3 College of Big Data and Internet, Shenzhen Technology University (SZTU), Shenzhen 518118, China

**Keywords:** machine learning, deep learning, detection, classification, multiscale features, features fusion

## Abstract

A brain tumor refers to an abnormal growth of cells in the brain that can be either benign or malignant. Oncologists typically use various methods such as blood or visual tests to detect brain tumors, but these approaches can be time-consuming, require additional human effort, and may not be effective in detecting small tumors. This work proposes an effective approach to brain tumor detection that combines segmentation and feature fusion. Segmentation is performed using the mayfly optimization algorithm with multilevel Kapur’s threshold technique to locate brain tumors in MRI scans. Key features are achieved from tumors employing Histogram of Oriented Gradients (HOG) and ResNet-V2, and a bidirectional long short-term memory (BiLSTM) network is used to classify tumors into three categories: pituitary, glioma, and meningioma. The suggested methodology is trained and tested on two datasets, Figshare and Harvard, achieving high accuracy, precision, recall, F1 score, and area under the curve (AUC). The results of a comparative analysis with existing DL and ML methods demonstrate that the proposed approach offers superior outcomes. This approach has the potential to improve brain tumor detection, particularly for small tumors, but further validation and testing are needed before clinical use.

## 1. Introduction

A brain tumor is an abnormal growth of cells in the brain, which can be either malignant or benign [1]. As the brain is a vital organ responsible for cognitive function, the presence of tumors can have life-threatening consequences. Brain tumors account for 85–90% of all central nervous system tumors, according to a report [2]. Radiologists use imaging techniques such as CT scans and MRI to locate cancer in the brain, with MRI providing higher-resolution imaging than CT scans. However, manually analyzing the images and grading tumors can be time-consuming, requires specialized expertise, and may still result in imprecise diagnoses and high costs [3]. These challenges arise from the asymmetrical shapes of tumors and the difficulty of distinguishing between different types of tumors that may look similar. Consequently, there is growing interest in developing computerized systems for recognizing brain tumors to improve diagnosis and treatment outcomes [4].

A computerized system utilizing traditional machine learning techniques has been created by researchers. The system involves a variety of processes, including preprocessing, feature extraction, dimensionality reduction, and categorization [5,6,7]. Feature extraction is the most important step in this process as it is necessary to automate brain tumor detection. However, the efficacy of this technique heavily relies on the type and nature of the features used, and traditional methods may not be able to identify small tumors in unseen samples or may work slowly when processing large amounts of brain MRIs. K-nearest neighbors (KNN), support vector machines (SVM), decision trees, and segmentation-based methods are some of the ML-based approaches used to address this problem. In the segmentation-based approach, algorithms are used to segment the tumor as a region of interest (ROI), followed by feature extraction from the ROI. This approach involves several steps, including preprocessing, finding a region of interest, feature extraction, training, and finally classification. However, due to the complex structure of the brain, the current techniques lack accuracy. Therefore, it is vital to develop an efficient and precise model for the timely detection of brain tumors with minimal human intervention [8].

Various popular deep learning models have been developed, including GoogleNet, InceptionNet, ResNet, VGGNet, DenseNet, and AlexNet. However, basic classification techniques used for detecting brain tumors only indicate the presence of a tumor and do not provide information about its location, leading to a high rate of false positives. Researchers have used different object detection methods for brain tumor identification to overcome this limitation. One study [9] utilized the deep learning-based CenterNet to localize brain tumors, using ResNet34 having an attention module as a base network. However, most object detection-based techniques for brain tumor identification require widespread hyperparameters and entail high computational costs. To address these challenges, this study proposes a new approach for early brain tumor recognition. In the first phase, the tumor is located using segmentation. In the second phase, ResNet-v2 and Histogram of Oriented Gradients (HoG), and a DNN are used to extract features from the segmented image. In the end, the features are combined, and a BiLSTM is trained for three classes including glioma, pituitary, and meningioma. The suggested model accurately identifies tumor locations and enhances the detection accuracy by employing essential features extracted from the segmented area.

The aim of this study is to create a strong framework utilizing MFO and Kapur’s thresholding-based segmentation, along with feature fusion, for the purpose of identifying and categorizing brain tumors using various MRI images. Additionally, the goal is to propose an efficient system that can accurately detect and locate tumors in MRI scans that have not been seen before. The study also aims to develop a technique that can identify small tumors in MRI scans for better early detection. The proposed system was extensively tested and the results show that it performs significantly well in terms of accuracy and robustness for early recognition and categorization of brain tumors. The remaining sections of the study are dedicated to discussing existing methods in Section 2, demonstrating the proposed technique in Section 3, evaluating the experiment in Section 4, and concluding the study in Section 5.

## 2. Related Work

Several researchers have explored machine learning and deep learning-based methods for medical diagnosis, as mentioned in references [10,11]. Some of these approaches utilize segmentation techniques, such as ensemble deep networks, which require training from the initial stage. To address this issue, some researchers have introduced a drop-out layer during the testing phase to recognize uncertainties in lesion identification [12]. Additionally, in reference [13], a CNN-based approach was proposed, and data augmentation was performed to improve the classification accuracy. The study used three datasets and achieved an accuracy of 98.43%. DL-based methods are increasingly essential in several image-processing applications, including medical diagnosis [14]. In another study [15], data augmentation was performed using patch rotation and extraction techniques on 3064 images, and CapsuleNet was utilized for recognizing and categorizing brain cancer into three classes. In reference [16], VGG16 and AlexNet were utilized to attain features from brain scans, and a feature fusion method was employed for binary classification. Finally, an SVM was used to categorize the images, achieving an accuracy of up to 96%. In reference [17], an encoder-based technique was used for categorization, with an accuracy of 98.5%.

Reference [18] used ResNet50 with additional layers for binary categorization of brain tumor images and attained an accuracy upto 97%. In reference [19], the BrainMRNet model was proposed, consisting of attention layers, residual stages, and hyper-column technique, achieving an accuracy of 96.05%. Reference [20] utilized transfer learning-based CNN and a multiple logistic regression method outperforming the existing techniques on three benchmarks. Sachdeva et al. [21] proposed a brain tumor detection method using SVM and artificial neural networks, combined with a genetic algorithm, achieving an accuracy of 91% and 94.9%, respectively. Tahir et al. [22] explored different approaches to increasing classification accuracy by employing edge detection, noise removal techniques, and contrast improvements achieving an accuracy of 86%.

In recent studies, different techniques have been suggested for brain tumor detection. Sarah et al. [23] utilized Harris Hawks optimized neural networks and varied types of layers for modifying the architecture. They preprocessed the images for noise removal and candidate region recognition to identify tumor regions. The proposed method achieved 98% accuracy using the Kaggle dataset. Aruna et al. [24] developed an approach using pretrained CNNs such as InceptionV3, ResNet50, and VGG19. They concatenated deep features extracted through CNNs using a two-stage strategy and reduced dimensions further using PCA for categorization. The results showed improved classification accuracy, but their approach increased computational complexity. In another study, Bakary et al. [25] employed the transfer learning concept to develop an automatic brain tumor classification technique using MR images of the brain. They used the AlexNet model for feature extraction and binary classification, achieving an overall accuracy of 99.62%. However, they did not classify the images into specific types of tumors.

The study by Sarmad et al. [26] proposes an automated system for brain tumor detection that employs several steps to achieve a high accuracy in classifying different types of brain tumors. The first phase involves using linear contrast stretching to identify edges in the sample. In the second phase, a DNN with 17 layers is designed for segmenting the tumor. This step aims to accurately identify the location and boundaries of the tumor within the brain image. In the third step, a modified version of the MobileNetV2 architecture is utilized for extracting features. Transfer learning is used to train the network, which involves adapting the pretrained model’s parameters to the specific task of brain tumor detection. Then, an entropy-based controlled mechanism is utilized with multiclass support vector machines (M-SVM) for feature selection. This step aims to identify the most relevant features for tumor classification. Finally, M-SVM is utilized for brain tumor categorization, which involves identifying glioma, meningioma, and pituitary images. The proposed system achieves a high accuracy of 97.47% and 98.92% for meningioma and pituitary images, respectively.

While several methods have been developed for brain tumor detection, early detection remains a significant challenge. Early detection is critical for effective treatment and improved patient outcomes. A detail of the existing model is shown in Table 1.

## 3. Methodology

**Figure 1:**
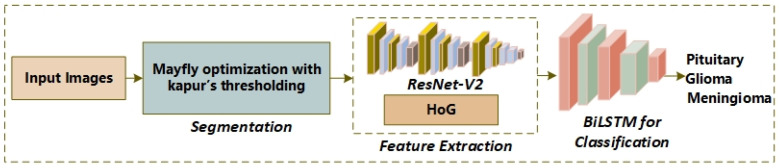
The flow diagram for the proposed model.

In this section, we introduce the working principals of the proposed model. The proposed system is a three-stage model as shown in Figure 1. The images of the brain are in grayscale; therefore, a preprocessing phase has been skipped. First, segmentation is employed using the mayfly optimization with a multilevel threshold approach. Second, the features are extracted from segmented tumors. Third, the brain samples are classified from the proposed multilayer perceptron (MLP).

### 3.1. MFO with Multi-Level Thresholding

*MFO* is one of the population-based methods developed in 2020 [28]. The concepts of *MFO* consist of the following functions; (1) initialization of equal number of male and female agents, (2) allowing the male mayfly to recognize the finest position as *loc* for the chosen task, (3) allowing the female mayfly to find and be merged with male mayfly located at *loc*, (4) offspring generation, and (5) termination of search and displaying the final output.

We employed a multilevel thresholding approach with *MFO* technique. Kapur et al. [29] proposed a threshold-based approach to compute the optimal thresholds for segmentation. The computation depends upon the distribution of probability and entropy of the image histogram. The approach determines the optimal threshold to maximize the entropy. For the bilevel threshold computation, an objective function can be attained as presented in Equation (1).

(1) $${FUN}_{kap}\left(t\right)=k_{1}+k_{2},$$

Here, $k_{1}$ and $k_{2}$ are computed as below:

(2) $$k_{1}=\sum_{s=1}^{t}\frac{p_{s}}{\omega_{0}}\ln\left(\frac{p_{s}}{\omega_{0}}\right)$$

(3) $$k_{2}=\sum_{s=t+1}^{L}\frac{p_{s}}{\omega_{1}}\ln\left(\frac{p_{s}}{\omega_{1}}\right)$$

Here, $p_{s}$ refers to the distribution of probability (DP) of the intensity level of grayscale; $\omega_{0}$ and $\omega_{1}$ presents the DP for the class labels $k_{1}$ and $k_{2}$ as described in Equations (2) and (3). This entropy-based approach is flexible enough for multilevel thresholding. Thus, it is necessary to split the images into *n* class labels using *n* − 1 threshold numbers. The objective value can be changes as shown in Equation (4).

(4) $${FUN}_{kap}\left(T\right)=\sum_{s=1}^{n}k_{s},$$

Here, *T* = [*t*_1_, *t*_2_, *t*(*n* − 1)] presents a vector consisting of several threshold numbers. The entropies are described separately with the respective threshold *t* value; therefore, Equation (5) has been modified for *n* entropy.

(5) $$k_{n}^{c}=\sum_{i=t_{n+1}}^{L}\frac{p_{i}}{\omega_{n-1}}\ln\left(\frac{p_{i}}{\omega_{n-1}}\right)$$

where, ($\omega_{0}^{},\omega_{1},\ldots .,\omega_{n-1})$ presents the probability occurrence for the *n* classes, and for the optimal threshold numbers, the *MFO* approach is utilized. The *MFO* technique is projected similarly to mating method and flighting feature of the mayflies [28]. The mayflies in swarms are recognized as female and male individuals. The male mayfly performs more robustly consequently improving the optimization process. The *MFO* approach modifies the position depending upon the location *loc_i_*(*t*) and velocity *velocity_i_*(*t*) at current round:

(6) $${loc}_{i}\left(time+1\right)={loc}_{i}\left(time\right)+{velocity}_{i}\left(time+1\right),$$

All female and male mayflies modify the location employing Equation (6) with respect to *time*. However, they utilize unique velocity modifying features.

#### Mating

The above half female and male mayflies pass through mating and generate children. The offspring are generated from the parents as denoted in mathematical form below:

(7) $${offspr}_{1}=P\times Male+\left(1-P\right)\times Female$$

(8) $${offspr}_{2}=P\times Female+\left(1-P\right)\times Male$$

**Figure 2:**
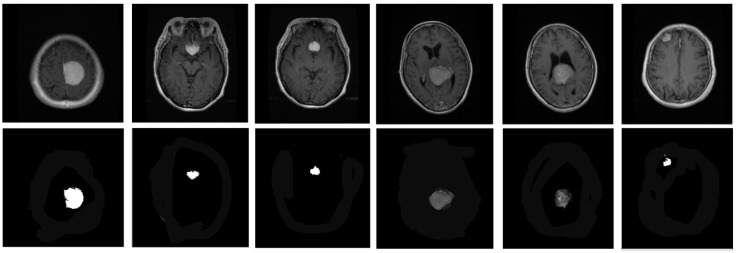
Some segmented samples from dataset. (**top**) Original images; (**bottom**) Segmented images.

Here, *P* refers the random numbers for Gauss distribution. Some segmented images are shown in Figure 2.

### 3.2. Features Extraction (FE)

The proposed approach involves utilizing two algorithms, ResNet-V2 and Histogram of Orientation Gradients (HOG), for feature extraction from brain images. ResNet-V2 is a deep neural network architecture that has been shown to be effective in image classification tasks, while HOG is a popular algorithm used for feature extraction in computer vision. After extracting features from the images, a classifier is trained using a Bi-directional Long Short-Term Memory (BiLSTM) network. BiLSTM is a form of recurrent neural network that can acquire lasting dependencies in sequential data. In this case, it is utilized to classify brain images into non-tumorous and tumorous classes based on the features extracted by ResNet-V2 and HOG. The use of deep learning techniques such as ResNet-V2 and BiLSTM has shown promising results in the field of medical image analysis, including brain tumor detection. The combination of different feature extraction algorithms can also improve the accurateness of classification, as different processes may capture different aspects of the image information.

#### 3.2.1. Histogram of Oriented Gradients (HOG)

This step involves extracting low-level features from tumor utilizing a Histogram of Oriented Gradients (HOG) algorithm. HOG is a popular feature extraction algorithm used in computer vision that captures the local gradient statistics of an image. In this approach, the segmented images are provided to a feature extractor block consisting of HOG and ResNet-V2, which is a deep neural network architecture. The HOG algorithm is utilized to extract a total of 1236 low-level features from the segmented images, using 9 bins to capture the gradient orientations. To improve the results, the intensity of the images can be improved by normalizing the images, although this is considered more valuable when the size of image is large.

To find the features, the images are first resized to compatible blocks of size 6 × 6 or smaller, and a stride of 4 is used for each 2 × 2-sized block. The HOG algorithm then computes the gradient magnitude and direction for each pixel in the image, with the direction ranging from 0–180 degrees. Pixels with similar orientations are grouped into the same bin, and the magnitude of the gradient for each pixel is computed using the mathematical equations. The magnitude *m* for the gradient of pixel (*i*,*j*) and the direction is attained as presented in below equations.

(9) $$m\left(i,j\right)=\sqrt[{2}]{i_{x}^{2}+j_{y}^{2}},$$

(10) $$\vartheta =\frac{i_{y}}{i_{x}}$$

where, $i_{x},i_{y}$ refers to the gradients in the directions of *x* and *y*. The ϑ exhibits the angle from 0 to 180.

#### 3.2.2. ResNet-V2

**Figure 3:**
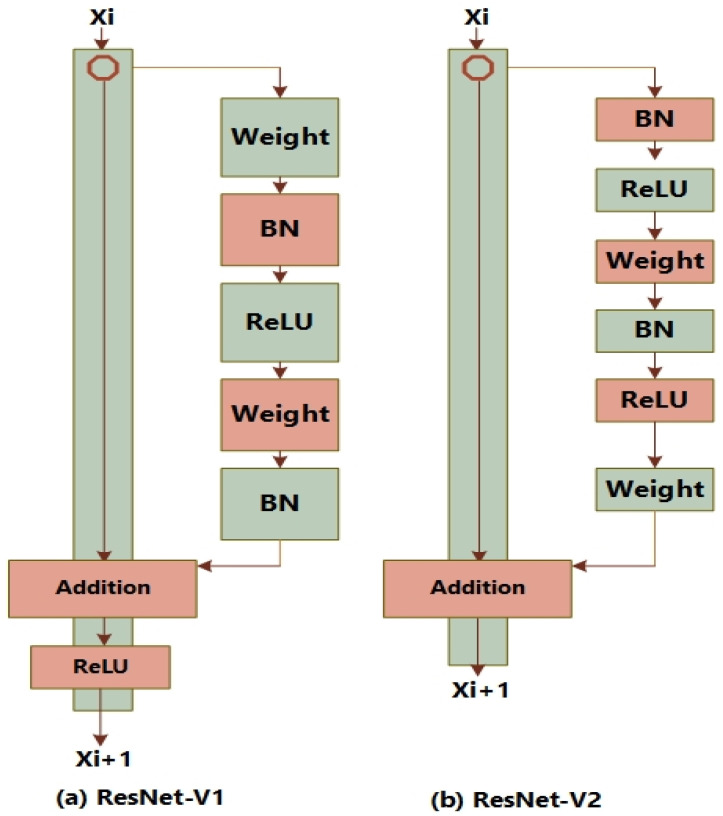
ResNet’s blocks.

He et al. [30] proposed ResNet and the block of residual, comprising two conv. layers and a connection for shortcut without any parameter that conveys the output of current block to the next block. The modification gave better performance than existing unmodified model in ILSVRC-2012 competition employing a 152 layered network and it was concluded that increasing the depth of the network results in improved classification accuracy. After the ResNet-V1, the authors improved the residual block so that ResLU function is not required in the shortcut connection, consequently increasing the detection accuracy. The main change in version 2 was implication of a stack as 1 × 1 batch normalization, 3 × 3 ReLU, and 1 × 1 2D convolutional layers. The architectures of ResNet-V1 and ResNet-V2 are shown in Figure 3.

### 3.3. Fusion Process

Feature fusion has been widely applied in various machine learning applications, including medical imaging [8]. It offers a dynamic approach to combining multiple feature maps, maximizing their integration. The model used for false positive detection relies on entropy. After obtaining the features, they are merged to form a single vector. Three vectors were computed as shown below:

(11) $$f_{Res\;1\times m}=\left\{{ResV2}_{1\times 1},{ResV2}_{1\times 2},{ResV2}_{1\times 3},\ldots \ldots \ldots ,{ResV2}_{1\times n}\right\},$$

(12) $$f_{HoG\;1\times D}=\left\{{HoG}_{1\times 1},{HoG}_{1\times 2},{HoG}_{1\times 3},\ldots \ldots .,{HoG}_{1\times n}\right\},$$

The fusion of features was utilized as presented below:

(13) $${Fusion(F{eat}_{vector})}_{1\times P}=\sum_{i=1}^{2}\{f{ResV1}_{1\times m}\;,\;{fHOG}_{1\times D}\},$$

Here, $\left\{{ResV1}_{1\times 1},{ResV1}_{1\times 2},{ResV1}_{1\times 3},\ldots \ldots \ldots ,{ResV1}_{1\times n}\right\}$ presents the feature vectors by ResNetV2, and $\left\{{HoG}_{1\times 1},{HoG}_{1\times 2},{HoG}_{1\times 3},\ldots \ldots .,{HoG}_{1\times n}\right\}$ presents the feature vectors by HOG. In this context, the feature vector f has undergone fusion. Then, an entropy value is calculated for the chosen features based on the specified value below.

(14) $$L_{he}=-N{he}_{b}\sum_{i=1}^{n}p\left(f_{i}\right),$$

(15) $$F_{sel}=L_{he}\left(\max\left(f_{i},1126\right)\right)$$

The probability of the features is denoted by p and their entropy is presented by $L_{he}$. The merged features are ultimately fed into the classifier to distinguish the samples with tumors.

### 3.4. Classification

At this stage, we present our classification model that was trained using the merged features to obtain optimal performance for detecting brain tumors. We utilized support vector machines (SVM), decision tree (DT), and our novel Bidirectional Long Short Term Memory (BiLSTM) to categorize the three categories of brain tumors. BiLSTM networks have been shown to provide better predictions than traditional LSTM networks, as they work in both forward and backward phases during training. The input features and weights are passed through multiple layers to generate the output, which is then used to compute the error. The parameters are adjusted during the backpropagation (BP) step to minimize the estimation errors. Furthermore, Bi-LSTM layers perform sequential function on input features. We set the hyperparameters as: ADAM optimizer, learning rate as 0.001, and a batch size 32. Our proposed network achieved the best detection results, followed by SVM, while the minimum detection accuracy was obtained using DT. The layers’ details are presented in Table 2.

## 4. Experimental Evaluation

Here, we explain the methods utilized for the performance assessment of the proposed method such as implementation details, protocols for training and testing, and several experiments.

### 4.1. Implementation Details

We utilized the several experiments employing a system integrated with a Graphical Processing Unit (GPU) card, i.e., NVIDIA (GE-FORCE GTX) integrated with 4 GB memory. The details of the environment are reported in Table 3.

### 4.2. Dataset

**Figure 4:**
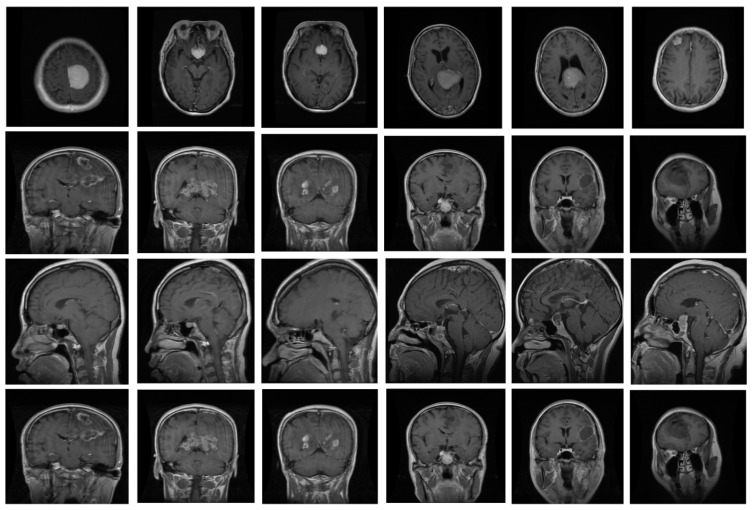
Some samples of brain MRI.

The proposed system underwent training and evaluation utilizing two distinct datasets: the Figshare dataset and Harvard medical images [31]. The Figshare dataset encompasses T1-weighted contrast-enhanced MRI images sourced from 233 individuals, yielding a total of 3064 brain images. This dataset consists of three categories of brain tumors, namely pituitary (930), glioma (1426), and meningioma (708), all of which were obtained from Nanfang hospital in China. The samples in this dataset measure 512 × 512 in size. On the other hand, the Harvard medical dataset was composed of ten tumors, as diagnosed by various experts. The brain MRIs in the Harvard dataset were captured in the axial plane, T2-weighted, and measured 256 × 256 pixels. We assessed the efficacy of our proposed detector using both datasets, but we only utilized the Figshare dataset for training purposes. Some sample images are depicted in Figure 4.

### 4.3. Metrics

The metrics used to evaluate the proposed model’s performance include precision, accuracy, recall, and *F*1 score. Below is the mathematical expression for these metrics.

(16) $$Precision=\frac{TP}{TP+FP},$$

The model’s accuracy is indicated by the correctly classified samples as per the proposed model, and this can be represented by Equation (17).

(17) $$Accuracy=\frac{TP+TN}{TP+TN+FP+FN},$$

The recall metric represents the proportion of diseased samples correctly identified by the proposed model, even if they were classified as noncancerous. The equations for recall and *F*1 score are presented below.

(18) $$Recall=\frac{TP}{TP+FN},$$

(19) $$F1\;score=2*\frac{Precision*Recall}{Precision+Recall},$$

The area under the curve (*Auc*) was found as below:

(20) $$Auc=\int_{i}^{j}f\left(x\right)dx,$$

### 4.4. Localization Results

The evaluation of our suggested segmentation method in this study is based on four parameters: DOI, TC, area, and number of pixels, which are expressed mathematically as follows:

(21) DOI=ω1+ω2,

(22) TC=∑x=1m∑y=1n(I′∩I)∑x=1m∑y=1n(I′∪I)

(23) $$area=\sum_{i=1}^{m}\sum_{j=1}^{n}I(i,j),$$

The TC quantity ranges from 0 to 1, and we evaluated segmentation methods using 80 images from each dataset and compared the findings by computing metrics from their corresponding ground truth images. The Harvard dataset’s ground truth images were assessed by a radiologist expert. Results for 12 images from the Harvard dataset are presented in Table 4, and those from the Figshare dataset are shown in Table 5. Table 6 presents a comparison of TC and DOI with other approaches over the Harvard dataset.

### 4.5. Classification Results

This experiment demonstrates the classification performance by our proposed approach on the Figshare and Harvard datasets. We used 500 images belonging to each class of the Figshare dataset for training our classifier, and 300 images from the same dataset for testing. Specifically, we tested 100 images for each of the three classes and achieved significant classification results as presented in Table 7. In addition, we trained three classifiers—decision tree (DT), SVM, and BiLSTM—and found that the BiLSTM network performed the best with an accuracy of 99.3%, a recall of 99.1%, precision of 98.3%, an F1 score of 99.1%, and an AUC of 0.989. For AUC computation, we considered the binary classes as pituitary vs all, glioma vs all, and meningioma vs all. Finally, we computed the average of all AUCs to determine the performance of each algorithm.

The highest classification results were obtained using SVM (98.3% accuracy) and then DT (97.3% accuracy) after implementing the BiLSTM. For the second trial, the Harvard dataset was utilized, consisting of 100 images from each of the three classes: pituitary, glioma, and meningioma. Our suggested BiLSTM achieved the best results, with 99.1% accuracy, 98.1% recall, 98.2% precision, 98.3% F1 score, and 0.974 AUC. After implementing our proposed classifier, the considerable classification results were achieved using DT, with an accuracy of 99.0%. The SVM classifier resulted in a minimum accuracy of 98.1% during cross-validation, as shown in Table 8.

### 4.6. Comparison with Existing Segmentation-Based Techniques

**Figure 5:**
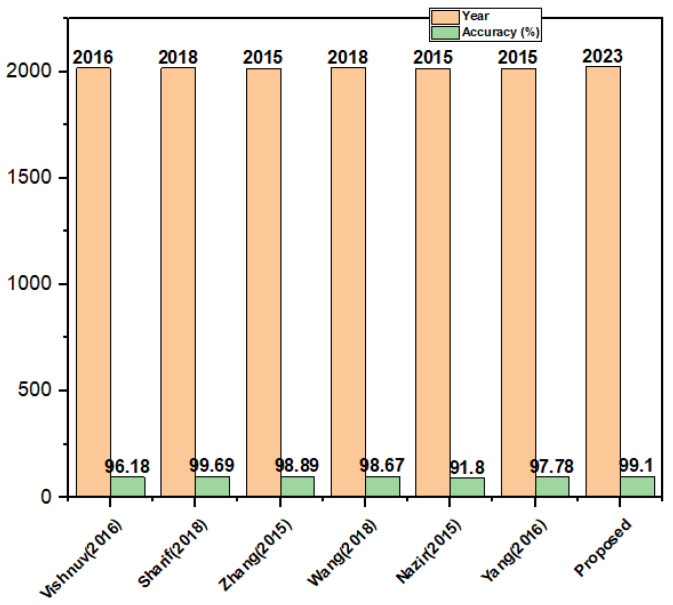
Comparison with existing methods on the Harvard dataset [32,33,34,35,36,37].

The objective of this experiment was to evaluate the effectiveness of our proposed MFO with a multilevel thresholding approach as a segmentation method. We conducted experiments in which we compared the performance of our method against several other established segmentation techniques including edge-based, region-based, multi-threshold, watershed, and otsu. We assessed the accuracy of the segmentation methods using the Harvard dataset and the results were presented in Table 9. Our experimental findings clearly demonstrate that our proposed segmentation-based method outperforms the traditional methods. A comparative plot illustrating the superiority of our segmentation-based method is presented in Figure 5.

### 4.7. Comparison with Existing DL-Based Techniques

**Figure 6:**
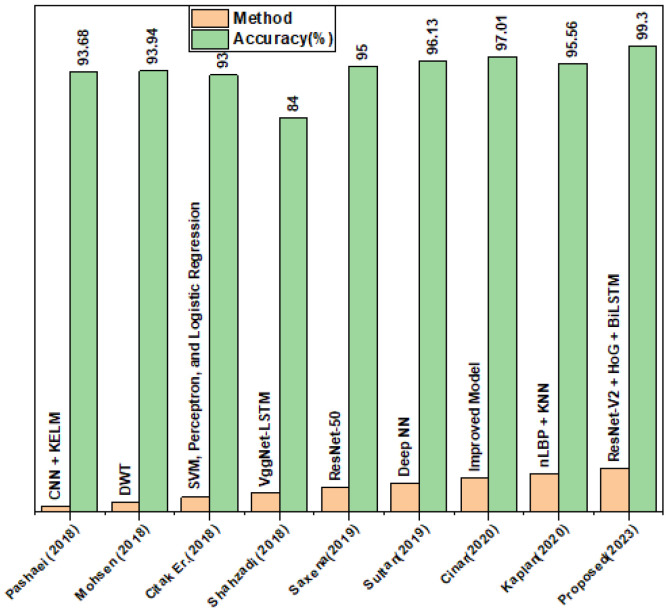
Comparison plot with existing techniques [18,39,40,41,42,43,44].

In this unit, we showcase various deep learning-based approaches for detecting and classifying brain tumors. We conducted an experiment to evaluate the accuracy of our proposed features fusion-based method compared to existing algorithms, and we reported the findings in Table 10. Our segmentation and features fusion-based approach performed exceptionally well, achieving an accuracy of 99.3%, which outperforms all other existing methods, including [26], which achieved the second-highest accuracy of 97.01%. In contrast, the lowest accuracy of 84% was obtained by [38], which utilized the VggNet-LSTM classifier. Our experiment clearly demonstrates that our proposed technique excels in segmentation, feature extraction, and brain tumor classification. Figure 6 displays the comparative plot of our results.

## 5. Conclusions

This study proposes a robust brain tumor detection method based on feature fusion that efficiently performs without requiring the preprocessing of brain samples. The proposed method involves segmentation using the mayfly optimization technique with multilevel thresholding for localizing tumors in brain MRI images. Features are extracted using HOG for local feature mining and ResNet-V2 for valuable feature mining. The merged features are then classified into three categories (pituitary, glioma, and meningioma) using a BiLSTM classifier. We trained and tested our model using the Figshare dataset and evaluated its robustness using the Harvard dataset. The proposed method achieved an accuracy of 99.3%, a recall of 99.1%, precision of 98.3%, an F1 score of 99.1%, and an AUC of 0.989, outperforming state-of-the-art segmentation and DL-based brain tumor detectors.

This automated method can be used directly by radiologists and oncologists to assist in the early detection of brain tumors. Additionally, the system provides precise tumor locations that can aid physicians in making surgical decisions.

Our approach has a limitation in terms of training time, which could be addressed using high computational systems. We also discovered that our system may have difficulty predicting the type of brain tumor when the MR images are blurry. To address these issues in the future, we aim to reduce the required time for training while maintaining the same level of performance. We also plan to add a preprocessing step to enhance images in situations where high-resolution imaging tools are unavailable. Furthermore, we intend to use our proposed method for detecting various cancers such as lungs, skin, and bone.

## Figures and Tables

**Table 1:** Details of some existing methods.

Sr. No.	Ref.	Type	Dataset	Issues	Advantages	Algorithm	Features	Performance
1	[12]	Segmentation	BraTs2021	High computational complexity is required.	Better results of segmentation	CNN-Transformer	CNN	93.50% Dice score
2	[26]	segmentation	Figshare and BRATS	Extra computational means are required.	Computationally efficient.	M-SVM	MobileNetV2	Accuracy: 98.92%
3	[23]	Classification	Kaggle	Minimum optimized feature selection.	Locates the tumor accurately.	CNN	CNN	Accuracy: 98%
4	[24]	Classification	3 benchmarks	High computational resources used.	Significant generalization.	Ensemble method	Xception, VGG19, EfficientNet, ResNet-50, and Inception-V3	Accuracy: 98.96%
5	[27]	Segmentation and classification	TCIA	Additional computational resources	Identifies tumor locations accurately.	Hybrid approach	Hand-crafted + CNN	Accuracy: 98.89%

**Table 2:** Layer information of the Bi-LSTM.

Type	Output Shape	Number of Parameters
Feature input	-	0
LSTM-1 (Forward pass)	100, 500	161,700
LSTM-2 (Backward pass)	100, 500	161,700
Max pooling layer	500	0
FC + ReLU	50	20,070
Dropout	50	0
FC (Sigmoid)	3	-

**Table 3:** Experimental environment details for the proposed model.

Hardware	Conditions
RAM	16 GB
Graphical Processing Unit	NVIDIA GEFORCE GTX × 4
Central Processing Unit	Intel Core i5
GPU Memory	4 GB

**Table 4:** The results on Harvard dataset of segmentation.

Image	TC (%)	DOI (%)	Pixels	Area (nm^2^)
Image 1	90	96	3244	7.1 × 10^11^
Image 2	92	97	2234	8.2 × 10^12^
Image 3	92	97	2321	8.3 × 10^11^
Image 4	96	98	3243	6.2 × 10^14^
Image 5	93	97	3421	6.3 × 10^11^
Image 6	90	94	2933	5.6 × 10^13^
Image 7	91	95	3284	7.2 × 10^12^
Image 8	97	99	4122	5.1 × 10^10^
Image 9	96	98	2847	3.5 × 10^12^
Image 10	98	98	4354	8.6 × 10^10^
Image 11	91	97	5121	7.0 × 10^12^
Image 12	98	99	2038	4.4 × 10^13^

**Table 5:** The results on Figshare dataset of segmentation.

Image	TC (%)	DOI (%)	Pixels	Area (nm^2^)
Image 1	91	93	2215	6.1 × 10^13^
Image 2	92	96	2225	5.1 × 10^12^
Image 3	93	96	3235	7.4 × 10^14^
Image 4	99	98	3357	5.4 × 10^13^
Image 5	93	97	3452	6.1 × 10^14^
Image 6	91	95	5312	6.8 × 10^13^
Image 7	92	95	4463	7.3 × 10^13^
Image 8	98	97	4471	6.1 × 10^14^
Image 9	99	99	3386	6.8 × 10^12^
Image 10	98	98	3496	6.8 × 10^13^
Image 11	0.99	98	2323	6.3 × 10^14^
Image 12	0.99	91	4344	7.3 × 10^13^

**Table 6:** The evaluation of results with Vishnuvarthanan et al. [32] using Harvard dataset.

Technique	TC (%)	DOI (%)
Graph Cut	27	43
SOM	23	37
SOM-FKM	31	47
FKM	22	36
Kernel	22	36
Our approach	99	97

**Table 7:** The classification results on Figshare dataset.

Algorithm	Accuracy (%)	Recall (%)	Precision (%)	AUC	F1 Score (%)
DT	97.3	96.8	97.2	0.900	97.2
SVM	98.3	98.1	98.9	0.912	98.5
BiLSTM	99.3	99.1	98.3	0.989	99.1

**Table 8:** The classification results on Harvard dataset.

Algorithm	Accuracy (%)	Recall (%)	Precision (%)	AUC	F1 Score (%)
DT	99.0	98.2	98.9	0.921	98.0
SVM	98.1	97.4	97.9	0.901	97.2
BiLSTM	99.1	98.1	98.2	0.974	98.3

**Table 9:** Comparison with state-of-the-art methods on Harvard dataset.

Algorithm	Year	Accuracy (%)
[32]	2016	96.18
[33]	2018	99.69
[34]	2015	98.89
[35]	2018	98.67
[36]	2015	91.8
[37]	2015	97.78
Proposed	2023	99.1

**Table 10:** Comparison with existing models based on deep learning.

Reference	Year	Method	Accuracy (%)
[39]	2018	CNN + KELM	93.68
[40]	2018	DWT	93.94
[41]	2018	SVM, Perceptron, and Logistic Regression	93
[42]	2018	VggNet-LSTM	84
[43]	2019	ResNet-50	95
[44]	2019	Deep NN	96.13
[18]	2020	Improved Model	97.01
[45]	2020	nLBP + KNN	95.56
Proposed	2023	ResNet-V2 + HoG + BiLSTM	99.3

## Data Availability

The datasets generated during and/or analyzed during the current study are available from the corresponding author on reasonable request.
